# Immune checkpoint inhibitor induced nephrotoxicity: An ongoing challenge

**DOI:** 10.3389/fmed.2022.1014257

**Published:** 2022-12-20

**Authors:** Martina Catalano, Giandomenico Roviello, Ilaria Camilla Galli, Raffaella Santi, Gabriella Nesi

**Affiliations:** ^1^School of Human Health Sciences, University of Florence, Florence, Italy; ^2^Department of Health Sciences, University of Florence, Florence, Italy; ^3^Histopathology and Molecular Diagnostics, Careggi Teaching Hospital, Florence, Italy

**Keywords:** immune checkpoint inhibitors, immune-related adverse events, nephrotoxicity, ICI-induced acute kidney injury, multidisciplinary management

## Abstract

Although immune checkpoint inhibitors (ICIs) have dramatically revolutionized the field of oncology over the last decade, severe immune-related adverse events (irAEs) are potentially life-threatening. In comparison with toxicities involving the skin, gastrointestinal tract and endocrine system, nephrotoxicity is less common but often underestimated due to difficult diagnosis. Management usually consists of treatment discontinuation and/or corticosteroid use. In this review, we summarize current knowledge of ICI-induced nephrotoxicity, evaluating drawbacks and future perspectives.

## 1. Introduction

The development of immunotherapy has revolutionized cancer treatment, allowing the possibility of long-term survival in patients with metastatic disease. Immune checkpoint inhibitors (ICIs) are monoclonal antibodies targeting checkpoint proteins expressed by immune cells or tumor cells, such as cytotoxic T lymphocyte–antigen 4 (CTLA-4), programmed cell death protein 1(PD-1), and PD-ligand 1 (PD-L1). Blockade of these molecules prevent tumor cells from escaping immune detection and reactivate cytotoxic T cells to recognize and destroy neoplastic cells (1, 2).

Notable improvement in overall survival (OS) and progression-free survival (PFS) in metastatic and advanced cancer patients, as well as benefits in early stages of the disease, have led the Food and Drug Administration (FDA) to approve ICI therapy for several cancers, including melanoma, non-small-cell lung cancer, urothelial cancer, and renal cell carcinoma (3–12). In some malignancies, immunotherapy has become the primary therapeutic choice replacing chemotherapy, however only one third of patients gain any benefit and two thirds experience adverse, occasionally fatal, events. These side-effects, defined as immune-related adverse events (irAEs), arise from autoimmune phenomena of varying degree of severity, potentially affecting all tissues. Cutaneous, gastrointestinal, and endocrine irAEs are more common and relatively easy to manage. Involvement of other organs, e.g., the kidneys, is less frequent and more difficult to diagnose and control (13). Acute kidney injury (AKI), usually resulting from acute interstitial nephritis caused by ICIs (ICI-AKI), occurs in a minority of patients (14). Electrolytic disorders, including hyponatremia, hypocalcemia, hypokalemia and Fanconi syndrome, call for vigilant monitoring to avoid life-threatening complications. Treatment of renal irAEs is based on the use of steroids and/or interruption of ICIs to prevent irreversible organ damage.

In this review, we summarize up-to-date information on the incidence, risk factors, and therapeutic strategies for ICI-AKI, evaluating future management perspectives.

## 2. Incidence of nephrotoxicity

Several systematic reviews and meta-analyses have evaluated the incidence of nephrotoxicity during ICI treatment (15–19) (Table 1). Common Terminology Criteria for Adverse Events (CTCAE) is widely accepted as the standard and severity grading scale for adverse events in cancer therapy. This system establishes five grades of AKI, depending on serum creatinine (sCr) elevation above the upper limit of the reference range (20). However, it should be borne in mind that cancer patients often have reduced muscle mass which can alter perception of any creatinine increase. Conversely, the Kidney Disease Improving Global Outcomes Work Group (KDIGO) consensus, defines three stages of AKI according to sCr modifications (21).

**Table 1:** Mains meta-analyzes evaluating the incidence of ICI-related AKI.

**Reference**	**No. of studies**	**ICI used**	**Phase of studies**	**No. of patients**	**Incidence**		
						**All grade**	**Grade ≥3**
Cortazar et al. (16)	4	Anti PD-1/PD-L1 (*n =* 3) or Anti CTLA-4/PD-1 (*n =* 1)	II e III	3,695	Total	2.2%	0.6%
					Mono therapy	0–0.9%	1.4–2.0%
					Combination	4.9%	1.7%
Abdel-Rahman et al. (17)	8	Anti-PD-1/PD-L1 (*n =* 6) or Anti-CTLA-4/PD-1 (*n =* 2)	II e III	4,070	Mono therapy	0.4–3.0%	0–1%
					Combination	2.1–6%	0–2%
Iacovelli et al. (15)	11	Aanti-PD-1 (*n =* 10) or Anti-CTLA-4/PD-1 (*n =* 1)	III	5,722	Total	1.4 % (0.4–3%)	0.2% (0–0.8%)
Wang et al. (18)	46	Anti-PD-1/PD-L1	I-III	12,808	Total	2%	1% (only with nivolumab)
Manohar et al. (19)	39	Anti-PD-1	II e III	11,482	Total	2.2%	19% of all grade

ICI, immune checkpoint inhibitor; AKI, acute kidney injury; PD-1, programmed death-1; PD-L1, programmed death-ligand 1; CTLA-4, cytotoxic T-lymphocyte antigen 4; Nr, number.

In a combined analysis of 3,695 patients receiving ICIs in phase II and III trials, the overall incidence of AKI was 2.2%, and the incidence of grade ≥3 AKI was 0.6% (16). A metanalysis of 4,070 patients showed that all grade nephrotoxicity risk was greater in patients treated with ICIs than those receiving chemotherapy, whereas no significant difference for high-grade AKI was recorded (17). The risk with both nivolumab and ipilimumab combination was higher than the risk with either ipilimumab (Odds Ratio [OR]: 0.47, 95% confidence interval [CI] 0.21–0.99) or nivolumab (OR: 0.11, 95% CI 0.03–0.29) alone.

A more recent meta-analysis of 5,722 patients included 10 clinical trials using ICI monotherapy (mainly anti-PD-1) and one study combining ipilimumab with nivolumab. Compared to controls, the incidence of anti-PD-1-related renal toxicity of all grades was significantly higher, reaching 1.4% (Relative Risk [RR]: 1.85, CI 95% 1.07–3.2), while the incidence of high-grade renal events was similar (0.1 and 0.2%, respectively) (15). The ICI-AKI incidence was not influenced by previous chemotherapy regimens, whereas pembrolizumab, but not nivolumab, correlated with a significant increased risk of developing renal toxicity of any grade (RR: 4.91; 95% CI 1.46–16.53; *p* = 0.01) (15). This could be explained by the greater susceptibility of patients affected by urothelial carcinoma and receiving pembrolizumab to develop renal injury.

Another meta-analysis conducted by Wang et al. evaluated the incidence of ICI-related nephrotoxicity (increased sCr, nephritis, and renal failure) in 46 trials comprising 12,808 patients administered anti-PD-1 or anti-PD-L1 monotherapy (18). The incidence of any grade nephritis was lower than 1%, while any grade and high-grade AKI was reported in about 2 and 1% of patients receiving nivolumab, respectively (18).

The most recent meta-analysis evaluated incidence of AKI, defined as an increase in creatinine ≥0.3 mg/dL from baseline, in 11,482 patients receiving anti-PD-1. In these patients treated with nivolumab or pembrolizumab, cumulative incidence was 2.2%, though this included all etiologies (19).

Although ICI-AKI appears to be infrequent, the risk can increase with ICI combined therapy (anti-CTLA-4 plus anti-PD-1/PD-L1 or ICI plus chemotherapy) (15, 18, 22). In a randomized phase III trial comparing platinum-based chemotherapy plus placebo or pembrolizumab, AKI (6.2 vs. 0.5%) and any grade (2 vs. 0%) or high grade (1.5 vs. 0%) nephritis was greater in patients treated with ICI than in those receiving placebo (22).

Finally, a recent real-world pharmacoepidemiology study of post-marketing surveillance data conducted by Chen et al., reported a gradually increase incidence of immune related renal adverse effects from 2011 to 2019 (23). Authors reported a larger number of renal adverse events with nivolumab monotherapy (33.24%), followed by combination therapy of nivolumab plus ipilimumab (23.55%) (23).

## 3. Pathophysiology, histological and clinical features

Anti-CTLA4 and anti-PD-1/PD-L1 reactivate the suppressor immune response through various mechanisms, which partly explain the different time of onset and grade of renal toxicity. By virtue of its higher affinity, CTLA-4 out-competes CD28 for ligand binding and blocks intracellular co-stimulatory signals, ultimately leading to inhibition of lymphocyte response to antigen presentation (24). Regulatory T lymphocytes (Tregs) lose their ability to suppress inflammation, activating an immune response against the tumor as well as healthy tissues and organs. As a consequence, increased renal lymphocyte infiltration may often occur in AKI during anti-CTLA-4 treatment.

PD-1 is a receptor expressed on various types of immune cells such as T and B lymphocytes, natural killer cells, monocytes and dendritic cells, and the interaction with its ligands (PD-L1 or PD-L2), at times expressed on cancer cells, leads to inhibition of effector T-cell activity (24). PD-1/PD-L1 pathway is pivotal in preventing inappropriate immune response in renal tissue, to the extent that kidney cells generally exhibit increased PD-L1 expression. Accumulating evidence suggests that PD-L1 hyperactivation prevents the development of autoimmune nephritis and glomerulonephritis (25–29).

The mechanisms by which ICIs stimulate autoimmune response may explain the different kinetics of ICI-mediated AKI manifestations. Renal damage caused by anti-CTLA-4 leads to early lymphocyte infiltration of renal tissue, with rapid onset averaging 6–12 weeks (16, 18). Conversely, anti-PD-1/PD-L1 treatment determines loss of tolerance and subsequent stimulation of immune response, resulting in nephrotoxicity onset at 3–12 months (16, 18).

Kidney injury can affect one or several compartments of the kidney (glomerulus, proximal/distal tubule, and interstitial tissue). Glomerular damage, including podocytopathy, membranous nephropathy and thrombotic microangiopathy, has been reported after administration of ipilimumab alone. Ipilimumab has also been associated with systemic lupus erythematosus-like nephritis, characterized by diffuse tissue damage and glomerular sclerosis (16, 18, 30, 31).

The use of anti-PD-1 and anti-PD-L1 has most frequently been linked to acute tubulo-interstitial nephritis, with diffuse tubulo-interstitial infiltrate of lymphocytes (mostly CD3+, CD4+), eosinophils and plasma cells. ICI-associated interstitial and tubular lesions may resemble lupus nephropathy and is generally associated with lymphocyte infiltrate and edema. Granulomatous inflammatory response, with or without tubular necrosis, have also been described during anti-PD-1/PD-L1 therapy (14, 17, 30–32).

Other types of kidney damage, such as IgA nephropathy and renal tubular acidosis, could also be related to ICIs. Thrombotic microangiopathy, a rare and potentially life-threatening adverse event, has recently been reported following ICI treatment (33). On suspicion of ICI-related AKI, renal biopsies have only rarely been performed and tissue damage has been poorly documented (33–35).

No clinical features reliably define AKI etiologies; however, some characteristics can be suggestive. Eosinophilia, although uncommon, may be of use (16, 19) while, sterile pyuria and subnephrotic-range proteinuria cannot confirm or rule out ICI-AKI (15, 16, 28). Notably, the latency period between ICI initiation and AKI onset is often longer than for other more commonly reported irAEs, and concomitant or prior extrarenal irAEs are all important clinical clues that should raise suspicion of ICI-AKI (16, 36, 37).

## 4. Diagnosis

The role of kidney biopsy in the diagnosis of ICI-AKI remains to be clarified. In the absence of any contraindication, some authors always recommend performing kidney biopsy to ascertain diagnosis, while others recommend only when a different etiology is suspected (e.g., acute glomerulonephritis) (38). The National Comprehensive Cancer Network does not recommend kidney biopsy unless grade ≥2 (39) while the American Society of Clinical Oncology (ASCO) recommends proceeding with steroid therapy without kidney biopsy, monitoring blood creatinine before each drug infusion, as well as urine analysis with proteinuria evaluation in case of acute kidney injury (40). Differentiating AKI due to ICI-therapy from another cause is a diagnostic challenge and overdiagnosis of irAE is undesirable as it would lead to unnecessary discontinuation or postponing of cancer therapy and side effects from steroid therapy. In the absence of an absolute contraindication and of any other potential causes of AKI (i.e., urinary tract infection, recent exposure to iodinated contrast medium, concomitant nephrotoxic drugs, dehydration, and obstructive causes) kidney biopsy would be quite helpful in guiding management. Indeed, clinical symptoms and laboratory tests are insufficient to differentiate the causes of ICI-associated AKI anda histological confirmation would be useful, to confirm acute interstitial nephritis or immune-mediated glomerulonephritis that require drug discontinuation and corticosteroid use, from non-immune mediated causes of AKI.

## 5. Management and rechallenge

A significant increase in sCr levels during ICI treatment should be considered indicative of immune-related nephritis until proven otherwise. After the most frequent causes of AKI have been excluded, depending on the grade of toxicity, specialized management and timely therapy initiation are highly recommended. Due to the low incidence of these adverse events, no controlled clinical studies have been designed to specifically evaluate outcomes of therapeutic management, and recommendations are consequently based on the major international guidelines, as summarized in Table 2 (40–42).

**Table 2:** Management of ICI-AKI in relation to toxicity grade.

**Grade**	**ICI**	**Monitoring**	**MDT**	**Renal biopsy**	**Treatment**	**Relapse/re-challenge**
1	Consider holding immunotherapy	Follow urine protein/Cr ratio every 3–7 days	Consider nephrology consult if Cr remains unchanged over 2 weeks	—	—	—
2	Hold immunotherapy	Follow urine protein/Cr ratio every 3–7 days	Nephrology consultation	Consider if feasible prior to starting steroids	Start prednisone 0.5-1 mg/kg/day if other causes are ruled out For persistent G2 beyond 1 week, prednisone/methylprednisolone 1–2 mg/kg/day	Consider on resolution to ≤ G1, concomitant with or without steroid if Cr is stable. If relapse: monitor Cr every 2–3 weeks or more frequently as clinically indicated. If Cr remains stable, consider longer durations between Cr checks.
3–4	Hold (definitively) immunotherapy	Consider inpatient care Follow urine protein/Cr ratio every 3–7 days	Nephrology consultation	Consider if feasible prior to starting steroids	Prednisone/methylprednisolone 1–2 mg/kg/day Consider adding one of the following if kidney injury remains >G2 after 4–6 weeks of steroids; azathioprine; cyclophosphamide; cyclosporine; infliximab; mycophenolate	For resolved G3 (to ≤ G1) renal irAE, may consider re-challenge if clinically indicated, at least after ≥2 months of holding ICI therapy

Cr, creatinine; MDT, multidisciplinary team; ICI, immune checkpoints inhibitors; G, grade.

Anyhow, treatment with high doses of prednisone (at least 1 mg/kg) should be administered for no more than 3 weeks or until complete recovery of baseline kidney function, followed by a tapering period of 5–6 weeks, as recent evidence reported (43). Moreover, a retrospective study reported that a further shorter duration of corticosteroids (28 days or less) for patients with ICI-associated AKI was similar to longer durations in regards of kidney function recovery and risk of recurrence (44). If steroid therapy shows no improvement, the ASCO guidelines suggest other immunosuppressive agents such as azathioprine, cyclophosphamide, cyclosporine, infliximab or mycophenolate (40). Retrospective studies and case reports have described the efficacy of mycophenolate 1 g twice daily in patients with steroid refractory irAEs, including those involving the kidney (45).

According to the ASCO guidelines, ICIs should definitively be discontinued in all patients who develop grade ≥3 AKI, even though this could well deprive them of a potentially life-saving therapy. Results achieved before ICI suspension, as well as available alternative treatments, should be considered. Although rechallenge ICI seems to be an active and feasible strategy (46), further studies to clarify the safety of rechallenge after ICI-AKI are mandatory to reconsider ICIs once renal injury is resolved or stabilized.

## 6. Risk factors for nephrotoxicity

When compared to patients with normal renal function, patients with pre-existing renal disease and mild to moderate renal impairment show no clinically important differences in ICI clearance, and no starting dose adjustment is required (47–50). ICIs have also an acceptable tolerability profile in patients with severe renal impairment (glomerular filtrate <30 ml/min or on dialysis) (22, 24, 26). However, a multidisciplinary approach is essential for optimal management of patients with chronic renal failure and undergoing ICI treatment.

As previously mentioned, ICI combination therapy is a known risk factor for all types of irAEs, including AKI (3, 51–53). Sise et al. recently stated that proton pump inhibitors increase the risk of ICI-AKI through former sensitization of T lymphocytes to ICIs (54).

Concerns over the higher risk of rejection in transplant patients receiving ICIs have led to the exclusion of this population from clinical trials. Limited data retrieved from case reports on the safety and efficacy of ICIs in kidney transplant patients show conflicting results (55–66). Kidney transplant rejection occurs in 33 and 52% of patients treated with ipilimumab or anti-PD-1 antibodies, respectively, and in 55% of patients receiving ipilimumab followed by anti-PD-1 (67). One case of rejection was related to the use of anti-CTLA-4 combined with anti-PD-1 agents (68). Conversely, one kidney transplant patient on immunosuppressive therapy (tacrolimus and prednisolone) was administered ipilimumab and nivolumab as the disease progressed, without developing rejection (16).

Time elapsed between transplant and the start of immunotherapy, as well as type of maintenance immunosuppressive therapy, should be considered to prevent rejection. Ongoing studies are exploring alternative immunosuppressive regimens capable of reducing the incidence of rejection in patients who are candidates for ICI treatment (69).

## 7. Electrolyte disorders due to ICIs

In addition to AKI, electrolyte disorders have been reported with the use of ICIs. According to the metanalysis by Manohar et al., hypocalcemia is the most frequent electrolyte abnormality associated with PD-1 inhibitors, with grade ≥3 occurring in 13% of patients and one case resulting in death (19). Conversely, Wanchoo et al. in their review of the Food and Drug Administration adverse event database found that hyponatremia is the most common electrolyte disorder (61.5%) in patients receiving ICIs (14). More recently, Seethapathy et al. showed that only 0.3% of severe hyponatremia were due to endocrinopathies and that the risk factors for developing severe hyponatremia were the use of anti-CTLA-4 monotherapy compared to anti-PD-1, use of diuretics, and cirrhosis, and non-White race (70).

Hypomagnesemia has been described as an irAE with a variable incidence depending on the type of ICI used (19). It has been particularly associated with pembrolizumab (up to 27%) and as consequence of grade ≥3 ipilimumab plus nivolumab related enterocolitis (19). Moreover, hypomagnesemia should be monitored as possible cause of hypocalcemia development, and its correction is fundamental for correction of hypocalcemia (71).

Further evidence of electrolyte abnormalities, including symptomatic hypocalcemia with ipilimumab and nivolumab as well as severe hypokalemia and low serum bicarbonate with nivolumab, have also been reported (72, 73). The electrolyte disorders were managed with ICI discontinuation or supplementation therapy (e.g., calcium, vitamin D, and thyroid hormones).

Two cases of acquired Fanconi syndrome (proximal renal tubular acidosis with phosphaturia, glycosuria, and amino aciduria) associated with ICIs have been described. In the first case, a patient with hepatocellular carcinoma developed Fanconi syndrome 8 months after nivolumab initiation. Discontinuation of nivolumab together with aggressive intravenous and oral replacement of deficient electrolytes were required (74). In the second case, a patient with non-small cell lung cancer suffered from immune-related hepatitis followed by Fanconi syndrome after 4 weeks of ipilimumab and nivolumab treatment. After ICI discontinuation and administration of corticosteroids and immunosuppressive drugs, renal function was restored (75). The mechanisms underlying development of Fanconi syndrome remain unclear but could be related to the toxic effect of ICIs on the proximal tubules.

## 8. Conclusions and future directions

Although AKI is a rare complication of ICI therapy, failure to diagnose may lead to potentially life-threatening conditions. Depending on the type of ICI, AKI can occur weeks or months after treatment initiation. In patients with severe toxicity (grade ≥2), discontinuation of ICIs and/or treatment with corticosteroids are recommended. In the absence of a renal biopsy, lack of sensitive or specific clinical features to reliably diagnose ICI-AKI calls for the development of non-invasive biomarkers (e.g., urinary, blood, and imaging-based biomarkers) to identify those patients who could safely be rechallenged after an episode of ICI-AKI. Finally, it must be remembered that severe electrolyte abnormalities may develop during ICI therapy that necessitate regular monitoring.

## Author contributions

Study concept and design, analysis, interpretation of data, and had full access to all the data in the study and takes responsibility for the integrity of the data and the accuracy of the data analysis: GR. Acquisition of data: GR and MC. Drafting of the manuscript: GR, MC, IG, and RS. Critical revision of the manuscript for important intellectual content and supervision: GN. All authors contributed to the article and approved the submitted version.
